# Application of Gray Markov *SCGM*(1,1)_*c*_ Model to Prediction of Accidents Deaths in Coal Mining

**DOI:** 10.1155/2014/632804

**Published:** 2014-11-04

**Authors:** Jian-yi Lan, Ying Zhou

**Affiliations:** School of Energy Science and Engineering, Henan Polytechnic University, Jiaozuo 454000, China

## Abstract

The prediction of mine accident is the basis of aviation safety assessment and decision making. Gray prediction is suitable for such kinds of system objects with few data, short time, and little fluctuation, and Markov chain theory is just suitable for forecasting stochastic fluctuating dynamic process. Analyzing the coal mine accident human error cause, combining the advantages of both Gray prediction and Markov theory, an amended Gray Markov *SCGM*(1,1)_*c*_ model is proposed. The gray *SCGM*(1,1)_*c*_ model is applied to imitate the development tendency of the mine safety accident, and adopt the amended model to improve prediction accuracy, while Markov prediction is used to predict the fluctuation along the tendency. Finally, the new model is applied to forecast the mine safety accident deaths from 1990 to 2010 in China, and, 2011–2014 coal accidents deaths were predicted. The results show that the new model not only discovers the trend of the mine human error accident death toll but also overcomes the random fluctuation of data affecting precision. It possesses stronger engineering application.

## 1. Introduction

Coal is an important basic energy and raw material in China, and it accounts for 70% of primary energy [1]. However, due to the complexity and particularity of the coal mine, safety accidents cannot be well controlled, and it still is the key factor to restrict coal production capacity. On the other hand, the influence of coal mine safety accidents, in particular major accidents, is extremely bad, which can create serious losses to people's life and property [2].

Mine production system is a complex system, and the combined effects on various factors lead to coal mine safety accidents. Many scholars and experts consider that human error or people's unsafe behavior is the main reason for coal mine safety accidents by analyzing cause of accidents, and it accounts for more than 90% in all coal mine safety accidents [3–5]. Coal underground mining is used as the primary mining method in China, comparing to the surface mining, there are too many affecting factors in the underground, and human factors are the most important affecting factors in these factors. According to HFACS analysis methods in the coal mine accident, human factors are relatively complex and changeable [6], so more affecting data are unknown; it is a gray system. Low prediction accuracy can be avoided due to historical data lack or inaccuracy by applying the Gray *SCGM*(1,1)_*c*_ model to predict the coal mine safety accidents. At present, main methods for predicting safety accident include experience model, regression model, and the gray prediction method. Ting et al. had predicted the highway accident by using the empirical model [7]. Xiao-fu and Ya-dong had applied regression model to forecast the ship traffic accident [8]. Using the regression models and empirical models to predict accident requires large amounts of historical data. However, accidents are mainly human error accident in the coal mine. Due to human error is affect by many factors, and in a dynamic time-varying system with low accident data and the non-line random changes, so it is not suitable to use these methods for prediction. In the gray accident prediction, Shan et al. had predicted mine safety accident use of unbiased gray model [9]. Meng and Cefeng had applied gray correlation on human error accident prediction in the nuclear power plant [10]. Mu-dan et al. and Rui-bo et al. had, respectively, applied the* GM(1, 1)* model and residual* GM(1, 1)* model to predict coal mine accidents [11, 12]. Appling the gray model to predict accident, the prediction effect and scope had been further improved [13, 14]. However, qualitative analysis intensity should not be impressive enough, and the prediction accuracy should be less if these gray models were individually applied. The main reason is that the model requires data sequence must be exponential distribution, and fitting will be poor when data sequence fluctuations are comparatively large.

Based on system cloud gray prediction model features in [15], combining the advantages of both Gray prediction and Markov theory, according to the coal mine accident deaths provided by the State Administration of Coal Mine Safety, referencing the literature [16, 17], an amended Gray Markov *SCGM*(1,1)_*c*_ model is proposed. The gray *SCGM*(1,1)_*c*_ model is applied to imitate the development tendency of the mine safety accident, and the amended model is to improve prediction accuracy while Markov prediction is used to predict the fluctuation along the tendency, so as to further improve the prediction accuracy on random volatile accident data.

## 2. Establishing Prediction Model

### 2.1. Gray *SCGM*(1,1)_*c*_ Model Established

#### 2.1.1. Model Selection

According to the actual accidents situation in coal mine, human error of the coal mines is not regular; there are some characteristics which include occurrences randomly scattered, raw data samples lack, and imperfect and uncertain information. Modeling is more difficult to use probabilistic statistical methods or mathematical statistics. Gray Markov *SCGM*(1,1)_*c*_ prediction model possesses these characteristic as less information required, easy calculation, high accuracy, and so on. It does not list factors data affecting research object but finds useful information and explores the inherent laws from their own time data sequence establishing model to predict. The Gray Markov *SCGM*(1,1)_*c*_ prediction model is the ideal model to forecast coal mine safety incidents.

#### 2.1.2. Data Processing

Taking account of randomness of human error data in mines, original time series *x* ^(0)^ of coal mine safety accident deaths can be expressed:

(1) $$\begin{array}{c} x^{\left(0\right)}=\left\{x^{\left(0\right)}\left(1\right),x^{\left(0\right)}\left(2\right),\ldots ,x^{\left(0\right)}\left(n\right)\right\}. \end{array}$$

First, *x* ^(0)^ is integrated as follows:

(2) x−(1)=x−12,x−13,…,x1n,here,  x−1(k)=∑m=2kx−0m, k=2,3,…,n.

${\overset{-}{x}}^{\left(0\right)}$ is a close mean value generated sequence for *x* ^(0)^:

(3) x−(0)=x−02,x−03,…,x−0n,here,  x−0k+1=x0k+1+x0k2.

#### 2.1.3. Response Function

Human error is random to lead accidents in coal mine, so majority of accidents are dynamic. Given that integral sequence of safety accident deaths time series is expressed as $\left\{{\overset{-}{x}}^{\left(1\right)}(k)\right\}$ that is associated with satisfaction trend of nonhomogeneous index discrete function as *f*_*r*_(*k*) = *be* ^*a*(*k*−1)^ − *c*, thus the data of ${\overset{-}{x}}^{\left(1\right)}(k)$ is fit to *f*_*r*_(*k*). According to gray system cloud forecast method, the system gray *SCGM*(1,1)_*c*_ prediction model can be expressed as

(4) $$\begin{array}{c} \frac{d{\overset{-}{x}}^{\left(1\right)}\left(k\right)}{dk}=a{\overset{-}{x}}^{\left(1\right)}\left(k\right)+U,\;k\geq 2. \end{array}$$

Its time response function can be expressed as

(5) $$\begin{array}{c} {\overset{-}{x}}^{\left(1\right)}\left(k\right)=\left[\left({\overset{-}{x}}^{\left(1\right)}\left(1\right)+\frac{U}{a}\right)\right]\cdot e^{ak}-\frac{U}{a}. \end{array}$$

Here,

(6) a=ln⁡∑k=3nx−(0)(k−1)x−(0)(k)∑k=3nx−0k−12,b=(n−1)∑k=2nea(k−1)x−(1)(k)−(∑k=2nea(k−1))(∑k=2nx−(1)(k))(n−1)∑k=2ne2a(k−1)−∑k=2neak−12,c=1n−1(∑k=2nea(k))b−∑k=2nx−(1)(k).

Given ${\overset{-}{x}}^{\left(1\right)}(1)=b-c$, *U* = *ac*, ${\overset{-}{x}}^{\left(1\right)}(k)$ is reverted, the system gray *SCGM*(1,1)_*c*_ prediction model of original data will be expressed as

(7) $$\begin{array}{c} {\hat{x}}^{\left(0\right)}\left(k\right)=\frac{2b\left(1-e^{-a}\right)}{1+e^{-a}}\cdot e^{a\left(k-1\right)}, \end{array}$$

(8) $$\begin{array}{c} Y\left(k\right)=\frac{x^{\left(0\right)}\left(k\right)}{{\hat{x}}^{\left(0\right)}\left(k\right)}, \end{array}$$

(9) $$\begin{array}{c} \varepsilon \left(k\right)={\hat{x}}^{\left(0\right)}\left(k\right)-x^{\left(0\right)}\left(k\right),\;\;\Delta k=\frac{\left|\varepsilon \left(k\right)\right|}{x^{\left(0\right)}\left(k\right)}. \end{array}$$

*Y*(*k*), *ε*(*k*), and Δ*k* are gray fitting accuracy indicators, which reflected the degree of deviation of the predicted values to the original data.

### 2.2. Establishment of the Residual Amended *SCGM*(1,1)_*c*_ Model

Statistics data fluctuations of coal mine accidents deaths are larger, and the regularity is not very strong as uncertainty of the person's behavior. Therefore, the prediction accuracy should not be too good if the *SCGM*(1,1)_*c*_ model is solely applied to predict accident deaths of coal mine. In order to improve the prediction rate and better meet the actual situation, the prediction model should be corrected to improve the accuracy. Amended principle and steps are as follows.

(1) The first time residuals data sequence is got in accordance with the predicted value and actual value:
  (10) ε0k=x^0k−x0k, k=1,2,…,n,
  (11) ε0k=ε01,ε02,…,ε0n.
(2) Processing residuals correction sequence.

Given *M* = (1 + *e* ^−*a*^)^−1^(1 − *e* ^−*a*^)*b*, the *SCGM*(1,1)_*c*_ prediction will be expressed as ${\hat{x}}^{\left(0\right)}(k)=2e^{a(k-1)}M$. If *ε* ^(0)^(*k*) ≥ 0, (*k* = 1,2,…, *n*), the residual amended *SCGM*(1,1)_*c*_ model will be expressed as ${\hat{\varepsilon }}^{\left(0\right)}(k)=2e^{a_{1}(k-1)}M_{1}$. *a*_1_, *M*_1_, and *b*_1_ can be obtained in accordance with *a*, *M*, and *b* used method. The first time residuals corrected *SCGM*(1,1)_*c*_ model can be expressed as follows:

(12) $$\begin{array}{c} {\hat{x}}_{\varepsilon_{1}}^{\left(0\right)}\left(k\right)=2\left(e^{a\left(k-1\right)}M-e^{a_{1}\left(k-1\right)}M_{1}\right). \end{array}$$

If *ε* ^(0)^(*k*) < 0, (*k* = 1,2,…, *n*), the first time residuals corrected *SCGM*(1,1)_*c*_ model can be expressed as follows:

(13) $$\begin{array}{c} {\hat{x}}_{\varepsilon_{1}}^{\left(0\right)}\left(k\right)=2\left(e^{a\left(k-1\right)}M+e^{a_{1}\left(k-1\right)}M_{1}\right). \end{array}$$

In general, a prediction model can be repeatedly corrected residuals, and the residuals can be negative, the time dimensions are also not equal. If the first time residuals amendment cannot meet the forecast accuracy required, it should do residual correction according to the above amended principles, until the accuracy meets requirements. The residual correction generic model can be expressed as follows:

(14) $$\begin{array}{c} {\hat{x}}_{\varepsilon_{j}}^{\left(0\right)}\left(k\right)=2\left(e^{a\left(k-1\right)}M\overset{n}{\underset{j=1}{\sum }}e^{a_{j}\left(k-1\right)}M_{1}\right). \end{array}$$

### 2.3. Establishment of Amended Markov *SCGM*(1,1)_*c*_ Model

The *SCGM*(1,1)_*c*_ prediction fitting curve is essentially an exponential curve, and the prediction result is a relatively smooth curve. Because human error accidents are main part of coal mine accidents, the *SCGM*(1,1)_*c*_ model solely applied cannot meet forecast accuracy requirements. Markov theory has no aftereffect, that is to say, “the future state of the system is only related to the current state, and has nothing to do with the past state.” Meanwhile, Markov model is adopted to predict states trends through probability transfers, it can adapt to the randomness and variability of state. Applying Markov theory to correct the *SCGM*(1,1)_*c*_ prediction model of coal mine accident deaths can better solve the variability and randomness of accidents caused by human errors to improve the prediction accuracy.

#### 2.3.1. State Divided

The annual change of the number of deaths in coal mine accidents is a dynamic nonstationary random process, and thus the prediction fitting precision indicators also are variability and randomness. Because boundary and connotation of the different annual state are changeable, an adaptive state divided criterion needs to be determined, and the criterion should be consistent with basic timing trend of the coal mine accident deaths. Thus, *Y*(*k*) was divided into *m* states, and each state can be expressed as

(15) $$\begin{array}{c} E_{i}\in \left[{⊗}_{1i},{⊗}_{2i}\right],\;i=1,2,\ldots ,m. \end{array}$$

Here, ⊗_1*i*_ = *Y*(*k*) + *A*_*i*_, ⊗_2*i*_ = *Y*(*k*) + *B*_*i*_.

In the formula, *E*_*i*_ is expressed as *i* state, ⊗_1*i*_ and ⊗_2*i*_ are, respectively, expressed as the upper and lower bounds of the *i* state, and *A*_*i*_ and *B*_*i*_ are constants determined according to prediction data. Because *Y*(*k*) is a time function, ⊗_1*i*_ and ⊗_2*i*_ will be changed with time, so the state possesses variability.

When the state is divided, the numbers of different intervals are reasonably divided according to the actual situation. If raw data are less, the interval division should be less so as to increase the number of transfers between the various states, and thus the transfer law can be more objectively reflected between states. Conversely, if raw data are more, the interval division should be less in order to excavate more information from a large number of data to improve the prediction accuracy. It is suitable to adapt clustering classification method to determine class number and classification intervals due to less data and uncertain status of human error accidents in the coal mine.

#### 2.3.2. Construction of State Transition Rate Matrix

The original number of samples is expressed as *M*_*ij*_(*k*) from the state *E*_*i*_ transiting to the sate *E*_*j*_ by *k* step, and the number of occurrences of the state *E*_*i*_ is expressed as *M*_*i*_, so state transition probability is expressed as follows:

(16) $$\begin{array}{c} P_{ij}\left(k\right)=\frac{M_{ij}\left(k\right)}{M_{i}},\;i,j=1,2,\ldots ,m. \end{array}$$

*m* × *m* state transition probability matrix can be obtained as follows:

(17) $$\begin{array}{c} P\left(k\right)=\left[\begin{array}{cccc} P_{11}\left(k\right) & P_{12}\left(k\right) & \cdots & P_{1m}\left(k\right)\\ P_{21}\left(k\right) & P_{22}\left(k\right) & \cdots & P_{2m}\left(k\right)\\ & & \vdots & \\ P_{m1}\left(k\right) & P_{m2}\left(k\right) & \cdots & P_{mm}\left(k\right) \end{array}\right]. \end{array}$$

#### 2.3.3. The Predictive Value Determined

The state transition probability matrix *P*(*k*) reflects all statistical regularities of state transition, and the future system state steering can be predicted by investigating the matrix. In the actual analysis of the process, one step transition probability matrix *P*(1) is generally only examined. Given that predicted moment object is in the state *E*_*k*_, investigating the *k* row of *P*(1) can get the following.

(1) If max⁡*p*_*ij*_ = *p*_*kl*_, the next time system should most likely shift from the state *E*_*k*_ to the state *E*_*l*_.
(2) If there are two or more probability values identical or similar to *k* row in the matrix *P*(1), the future state steering will be difficult to determine; it needs to consider probability transition matrix *P*(2) or *P*(*n*) (*n* ≥ 3).

The system's future state will be determined by investigating state transition probability matrix, and gray change interval of relative prediction value in the future moments also will be determined; it can be expressed as [⊗_1*i*_, ⊗_2*i*_]. Predicted value of the future moment can be expressed as the interval median as *Y*′(*k*):

(18) $$\begin{array}{c} Y^{\prime }\left(k\right)=\frac{x^{0}\left(k\right)}{{\hat{x}}^{\prime 0}\left(k\right)}=\frac{1}{2}\left({⊗}_{1i}+{⊗}_{2i}\right)=Y\left(k\right)+\frac{1}{2}\left(A_{i}+B_{i}\right). \end{array}$$

## 3. Forecast Instances

**Figure 1:**
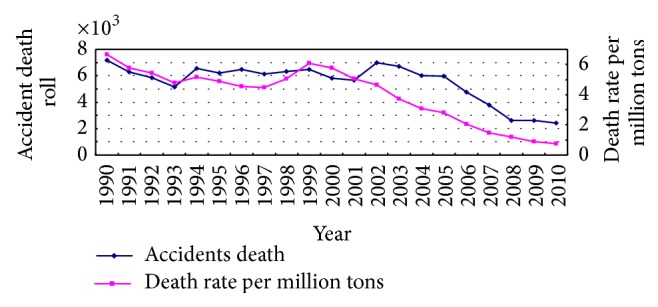
The statistics of megaton death rate and toll from 1990 to 2010 in China.

Chinese coal mining is primarily underground mining; the main mining methods used include blasting mining, general mechanized mining, and comprehensive mechanized mining from 1990 to 2010. Different methods lead to frequency of the coal mine accidents is not same. In general, blasting mining and general mechanized mining were usually applied in the town local small coal mine; their production capacity is relatively small and mining technology and management level are relatively lower compared to large coal mines, so accidents rate is high. In contrast to these small coal mines, the large state-owned coal mines mainly used comprehensive mechanized mining technology; their production capacity is relatively large, management level is relatively advanced, and the accidents rate is lower. Over the last decade, due to the small and medium sized coal mines integrated and strengthened security management in China, their production capacity and mining technology were gradually improved, so the number of occurrences of accidents in the coal mine was declined. According to statistics data of 1990–2010 coal mine accident death provided by Coal Mine Safety Administration, the trend of accidents deaths in coal mine can be fitted by applying the gray *SCGM*(1,1)_*c*_ model and prediction accuracy can be improved by the residual modified model. Finally, deaths are validly predicted by using the gray Markov *SCGM*(1,1)_*c*_ model. The raw data are shown in Table 1, the trend of coal mine accident deaths and death rate per million ton from 1990 to 2010 are shown in Figure 1.

### 3.1. The Establishment of *SCGM*(1,1)_*c*_ Model

*SCGM*(1,1)_*c*_ model of coal mine accident death toll is established by using accidents mortality data of coal mines from 1990 to 2010 in China:

(19) $$\begin{array}{c} {\hat{x}}^{\left(0\right)}\left(k\right)=\frac{2b\left(1-e^{-a}\right)}{1+e^{-a}}\cdot e^{a\left(k-1\right)}. \end{array}$$

In the prediction model (19), *a* and *b* are the most important parameters; they are mainly dependent on the raw data to reflect the development trend of data. Here, coal mine accident deaths for 20 years in China were taken as raw data to predict, in order to find the trend of coal mine deaths. Therefore, it is important to select the sample data; according to the formulas (5) and (6), *a* and *b* can be calculated. Here, *a* = −0.039436, *b* = −236878.

Applying the formula (19), prediction value of coal mine safety accident deaths can be got, and then the gray fitting accuracy indicators also can be calculated by the formula (8), which reveal overall development trend of safety accident death toll in coal mine. Specific forecast and actual values are shown in Table 1.

### 3.2. Residuals Corrected *SCGM*(1,1)_*c*_ Forecast

In accordance with Table 1, according to (10), the residual initial sequence can be attained, and then one correction residual prediction model can be expressed as

(20) $$\begin{array}{c} {\hat{\varepsilon }}^{\left(0\right)}\left(k\right)=2e^{a_{1}\left(k-1\right)}\frac{1-e^{a_{1}}}{1+e^{a_{1}}}b_{1}. \end{array}$$

Here, *a*_1_ = −0.0632, *b*_1_ = −21827.6.

According to the formula (14), a residual modification prediction value of safety accident deaths can be obtained, and the results are shown in Table 2.

### 3.3. The Residual Modification Markov *SCGM*(1,1)_*c*_ Forecast

Comparing Tables 1 and 2, it is shown that forecast fitting accuracy of the *SCGM*(1,1)_*c*_ model has been improved after residual modification, but volatility of the gray fitting accuracy indicators is larger from analyzing Table 2. In order to solve this question, it needs to adopt Markov chain prediction for further enhancing accuracy and lowering the volatility. The prediction fitting indicators data in Table 2 are divided to 4 states by hierarchical clustering, and the results are shown in Table 3. The corresponding states of each year can be determined according to the divided states, and applying the formula (16), the state transferred introduction matrix can be obtained, and the first step state transition matrix is shown as follows:

(21) $$\begin{array}{c} P\left(1\right)=\left[\begin{array}{cccc} 1 & 0 & 0 & 0\\ 0 & 0 & 0 & 1\\ \frac{1}{6} & \frac{1}{6} & \frac{1}{2} & \frac{1}{6}\\ 0 & \frac{1}{10} & \frac{3}{10} & \frac{3}{5} \end{array}\right]. \end{array}$$

The future coal mine accident deaths toll can be predicted based on the above state transition probability matrix. accident deaths in 2003 year be in the state* E*_*4*_, according to the state transition probability method, examining the state transition probability matrix *P*(1) line 4 can get

(22) $$\begin{array}{c} \max p_{4j}=p_{44},\;j=1,2,3,4. \end{array}$$

After a year transition, under the control of coal mine accidents, the death toll of coal mine accidents in 2004 was most likely in the state* E*_*4*_. According to Table 3, interval of the state* E*_*3*_ is [1.05,1.2], that is, ⊗_13_ = 1.05 and ⊗_12_ = 1.2. Because ⊗_1*i*_ = *Y*(*k*) + *A*_*i*_, ⊗_2*i*_ = *Y*(*k*) + *B*_*i*_, and *Y*(*k*) = 1.19 in 2004 according to Table 2, *A*_*i*_ = ⊗_13_ − *Y*(*K*) = 1.05 − 1.19 = −0.14, *B*_*i*_ = ⊗_23_ − *Y*(*k*) = 1.2 − 1.19 = 0.01. Using the gray Markov *SCGM*(1,1)_*c*_ model, the death toll of coal mine accidents in 2008 is most likely expressed as

(23) Y′k=YK+12Ai+Bi=1.19+12−0.14+0.01=1.125,x′02004=x^′02004·Y′k=5433×1.125≈6112.

In general, in order to facilitate the calculation, the above equation can also be expressed as

(24) x′02004=x^′02004·12⊗13+⊗23=5433×121.05+1.20=6112.

Similarly, according to the formula (21) and Table 2, accidents deaths of coal mine from 2004 to 2013 can be predicted; prediction error comparison of the amended residuals *SCGM*(1,1)_*c*_ model and the amended residuals Markov *SCGM*(1,1)_*c*_ is shown in Table 4.

**Figure 2:**
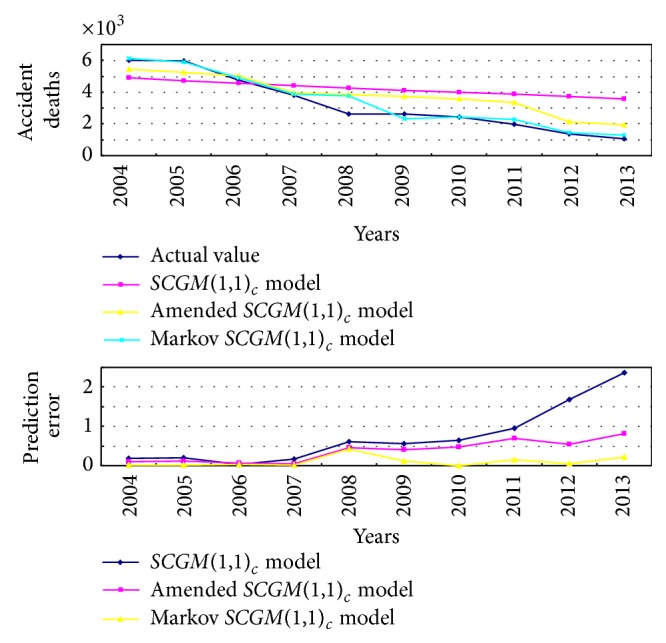
Curve fitting about coal mine death toll of different prediction models and error analysis chart.

Error analysis and predictive value fitting of three methods are shown in Figure 2.

Comparing Tables 1, 2, and 4, we know that Table 2 was amended by using residuals prediction model on the basis of Table 1, and the prediction accuracy had been appropriately improved. For Table 4, comparing the residual amended model and Markov prediction model can be seen that the residual amended *SCGM*(1,1)_*c*_ model cannot handle abnormal events, because its prediction parameters are fixed. As China's coal enterprises are affected by the economic situation form abroad and home, their production had been in low period, and some coal was not continuously produced; they were basically in an abnormal state in the last three years, so these factors would have a big impact on mine accident forecast. National and local government paid more attention to safety management in the coal mine, and each coal mine strengthened management and prevention on human error, they would also affect the accuracy of general forecasting methods. However, the Markov *SCGM*(1,1)_*c*_ prediction model is only depend on the previous state, the relationship with other states is very small, so it very better solves the abnormal accident. Its prediction error is significantly smaller than that of the amended *SCGM*(1,1)_*c*_ model. On the other hand, comparing accidents deaths of coal mine from 2004 to 2013 in China, the number of deaths was 6027 in 2004, and this figure became 1067 in 2013; in the recent ten years, the average decline rate of accidents deaths in coal mine is 16.8%. According to this downward trend, accidents deaths of coal mine in China will be about 889 in 2014.

As Figure 2 shows that the fitting degree of the Markov *SCGM*(1,1)_*c*_ model is the best, the amended *SCGM*(1,1)_*c*_ is better and *SCGM*(1,1)_*c*_ model is the worst. While error change of the Markov *SCGM*(1,1)_*c*_ is minimal, it illustrates that the Markov *SCGM*(1,1)_*c*_ forecast model can be used as a good method to predict the accident deaths of coal mine in China comparing to the other two methods; its prediction result for accident deaths of coal mine is basically corresponded with deaths decline trend in nearly 10 years.

## 4. Conclusions

The prediction gained by applying the amended residuals Markov *SCGM*(1,1)_*c*_ model is closer to the actual value; in other words, its error is smaller, and it can better reflect the relationship between the coal mine safety accident death and the number of data series, so the prediction is reliable. The amended residuals Markov *SCGM*(1,1)_*c*_ model combines the advantages of both the single-factor system cloud gray model and Markov chain, using residual to amend the system gray cloud; it can take full advantage of the information given by the historical data of coal mine accidents, and overcome the data random volatile effects on prediction precision.

## Figures and Tables

**Table 1:** Prediction and comparison of the coal mine accident deaths from 1990 to 2010 in China.

Year	Actual value	Prediction value
1990	7185	0
1991	6269	7716
1992	5854	7451
1993	5152	7196
1994	6574	6949
1995	6222	6710
1996	6496	6479
1997	6141	6257
1998	6304	6042
1999	6478	5835
2000	5798	5635
2001	5670	5441
2002	6995	5254
2003	6702	5074
2004	6027	4899
2005	5986	4731
2006	4746	4569
2007	3786	4412
2008	2631	4261
2009	2631	4114
2010	2433	3987

**Table 2:** Prediction and comparison of the coal mine accident deaths from 1990 to 2010 in China.

Year	Actual value	Amended prediction value	Actual value/prediction value
1990	7185		
1991	6269	6501	96%
1992	5854	6310	93%
1993	5152	6125	84%
1994	6574	5944	111%
1995	6222	5766	108%
1996	6496	5565	88%
1997	6141	5425	113%
1998	6304	6823	93%
1999	6478	6568	119%
2000	5798	6323	118%
2001	5670	6087	111%
2002	6995	5860	114%
2003	6702	5643	119%
2004	6027	5433	111%
2005	5986	5233	114%
2006	4746	5040	94%
2007	3786	3970	95%
2008	2631	3846	69%
2009	2631	3725	71%
2010	2433	3592	68%

**Table 3:** State division of deaths prediction for coal mine.

No.	Divided state	Actual value/prediction value
*E*_1_	Strong decreasing year	60%–75%
*E*_2_	Poor decreasing year	75%–90%
*E*_3_	Poor increasing year	90%–105%
*E*_4_	Strong increasing year	105%–120%

**Table 4:** Prediction comparison of deaths from 2004 to 2013.

Year	Actual value	Amended *SCGM*(1,1)_*c*_ model		Amended Markov *SCGM*(1,1)_*c*_ model	
		Prediction value	Relative error	Prediction value	Relative error
2004	6027	5433	0.099	6112	0.014
2005	5986	5233	0.126	5887	0.017
2006	4746	5040	0.062	4914	0.035
2007	3786	3970	0.049	3870	0.022
2008	2631	3846	0.462	3749	0.425
2009	2631	3725	0.416	2328	0.115
2010	2433	3592	0.476	2424	0.004
2011	1973	3357	0.701	2265	0.147
2012	1384	2142	0.548	1446	0.045

2013	1067	1934	0.813	1305	0.223
